# Phenotypic Similarity of Transmissible Mink Encephalopathy in Cattle and L-type Bovine Spongiform Encephalopathy in a Mouse Model

**DOI:** 10.3201/eid13112.070635

**Published:** 2007-12

**Authors:** Thierry Baron, Anna Bencsik, Anne-Gaëlle Biacabe, Eric Morignat, Richard A. Bessen

**Affiliations:** * Agence Française de Sécurité Sanitaire des Aliments–Lyon, Lyon, France; † Montana State University, Bozeman, Montana, USA

**Keywords:** prion, BSE, BASE, L-type, TME, mink, scrapie, research

## Abstract

Transmissible mink encepholapathy (TME) is a foodborne transmissible spongiform encephalopathy (TSE) of ranch-raised mink; infection with a ruminant TSE has been proposed as the cause, but the precise origin of TME is unknown. To compare the phenotypes of each TSE, bovine-passaged TME isolate and 3 distinct natural bovine spongiform encephalopathy (BSE) agents (typical BSE, H-type BSE, and L-type BSE) were inoculated into an ovine transgenic mouse line (TgOvPrP4). Transgenic mice were susceptible to infection with bovine-passaged TME, typical BSE, and L-type BSE but not to H-type BSE. Based on survival periods, brain lesions profiles, disease-associated prion protein brain distribution, and biochemical properties of protease-resistant prion protein, typical BSE had a distint phenotype in ovine transgenic mice compared to L-type BSE and bovine TME. The similar phenotypic properties of L-type BSE and bovine TME in TgOvPrP4 mice suggest that L-type BSE is a much more likely candidate for the origin of TME than is typical BSE.

## Abstract 2

L-type BSE is a more likely candidate for the origin of TME than typical BSE.

Transmissible mink encephalopathy (TME) is a rare prion disease in ranch-raised mink (*Mustela vison*) in North America and Europe (*1*–*4*). Six outbreaks have been reported from 1947 through 1985 in North America, and several have been linked to contaminated commercial feed (*1*). Although contamination of feed with scrapie-infected sheep parts has been proposed as the cause of TME, the origin of the disease remains elusive. The idea that scrapie in sheep may be a source of TME infection is supported by findings that scrapie-infected mink have a similar distribution of vacuolar pathologic features in the brain and the same clinical signs as mink with natural and experimental TME (*5*). However, mink are not susceptible to scrapie infection following oral exposure for up to 4 years postinoculation, which suggests that either the scrapie agent may not be the source of natural TME infection or that only specific strains of the scrapie agent are able to induce TME (*6**,**7*)*.*

Epidemiologic investigations in the Stetsonville, Wisconsin, outbreak of TME in 1985 suggested a possible cattle origin, since mink were primarily fed downer or dead dairy cattle but not sheep products (*8*). Experimental transmission of Stetsonville TME into cattle resulted in transmissible spongiform encephalopathy (TSE) disease with an incubation period of 18.5 months. Back passage of bovine TME into mink resulted in incubation periods of 4 and 7 months after oral or intracerebral inoculation, respectively, which was similar to that found following inoculation of Stetsonville TME into mink by these same routes (*8*). These findings indicated that cattle are susceptible to TME, and that bovine-passaged TME did not result in a reduced pathogenicity for mink. These studies raised the question as to whether an unknown TSE in cattle was the source of TME infection in the Stetsonville outbreak. Several additional TME outbreaks in the United States have been associated with mink diet that contained downer or dead cattle (*9*). These TME outbreaks happened before bovine spongiform encephalopathy (BSE) was identified in Europe or before 3 cases of BSE had occurred in the United States after 2003. Despite a potential link of TME with a cattle TSE, clinical and histologic studies indicate that mink inoculated with BSE have features that distinguish this disease from natural and experimental TME (*7*,*8*,*10*).

Recent studies demonstrate, on the basis of the molecular features of the protease-resistant prion protein (PrP^res^), that BSE has at least 3 different phenotypes in cattle (*11*). The cattle TSEs include: a) typical BSE, which is the prion strain identified during the BSE epidemic in Europe during the 1980s; b) H-type BSE, which is an uncommon type that was originally found in France (*12*); and c) L-type BSE, also called bovine amyloidotic spongiform encephalopathy (BASE), which is a rare form of BSE first identified in Italy (*13*). More recently, the H-type and L-type BSEs have been shown to differ from typical BSE with respect to incubation periods, vacuolar pathologic changes in the brain, and biochemical properties of PrP^res^ in mice on transmission into wild-type or transgenic mice that express the bovine prion protein gene (*14*–*18*). The origin of these BSE cases is unknown, but researchers have proposed that they represent a spontaneous form of TSE in cattle that is distinct from typical BSE; alternative hypotheses have also been considered, for example, cross-species contamination by another TSE source such as scrapie (*15*). If TME were to be due to infection with a cattle TSE, then these unusual forms of BSE are potential candidate agents since they may have a more widespread geographic distribution and were likely to precede the appearance of BSE in the USA.

In previous reports (*19**–**22*)*,* to compare typical BSE and scrapie strains ruminants TSEs were transmitted into transgenic mice (TgOvPrP4) that overexpress the ovine prion gene (*19*–*22*). In the current study, we investigated the possible origins of TME by transmission of the bovine-passaged Stetsonville TME agent into TgOvPrP4 mice, to compare the molecular and histopathologic features to those of typical and atypical BSE types. Our findings indicate that bovine TME is distinct from typical BSE and H-type BSE but shares many features with L-type BSE. The implications of these findings with respect to the origin of TME are discussed.

## Materials and Methods

### Cattle TSE Isolates

Cattle TSE isolates used in these studies included: a) the Stetsonville TME isolate experimentally-passaged into cattle as previously described (*8*); b) an L-type BSE isolate (02-2528) (*11*,*13*,*23*,*24*); c) a H-type BSE isolate (03-2095) (*12*,*14*,*17*); and d) a typical BSE isolate (01-2281), for which transmission in TgOvPrP4 ovine transgenic mice was previously described (*20*). The typical, L-type, and H-type BSE cases were diagnosed after active surveillance by rapid tests at rendering plants; the animals were 4, 8, and 12 years of age at death, respectively. For a comparison of BSE transmission without a species barrier in ovine transgenic mice, a BSE isolate passaged in sheep (SB1) was also included in mouse transmissions studies (*20*,*21*).

### Mouse Lines and Experimental Infections

The TgOvPrP4 mouse line expresses the ovine prion protein gene (A_136_R_154_Q_171_ genotype) in a PrP null mouse background as previously described (*25*). The ovine prion protein gene is 2–4× more important in a sheep brain of the same PrP genotype. Mice were cared for and housed according to the guidelines of the French Ethical Committee (decree 87-848) and European Community Directive 86/609/EEC. Experiments were performed in the biohazard prevention area (A3) of the author’s institution with the approval of the Rhône-Alpes Ethical Committee for Animal Experiments.

Female mice, 4 to 6 weeks of age (6–12 animals per experimental group), were inoculated intracerebrally with 10% (wt/vol) cattle brain homogenates in 5% glucose (20 μL per animal). Brain specimens from TgOvPrP4 mice in which a TSE developed were subsequently passaged by intracerebral inoculation of a 1% (wt/vol) homogenate into a second group of transgenic mice. Mice were sacrificed at the terminal stage of disease, and the brains were collected and either analyzed for PrP^res^ by Western blot or fixed in buffered 4% paraformaldehyde for histopathologic studies. Statistical analyses of survival periods were performed by using the log-rank test and the R software package (version 2.4.1); p values <0.05 were considered statistically significant.

### Western Blot Analyses of PrP^res^

PrP^res^ was extracted from bovine brain stem samples by using the TeSeE Western blot Bio-Rad kit (Bio-Rad, Marnes-la-Coquette, France; Ref 355 1169) following the manufacturer’s instructions. Briefly, 250 μL of 20% brain homogenates were incubated with an equal volume of reconstituted proteinase K solution (reagent A + PK) at 37°C for 10 min. After addition of 250 μL of reagent B, samples were centrifuged at 15,000× *g* for 7 min. The pellets were resuspended in 50 μL of denaturing buffer (TD4215) (4% sodium dodecyl sulfate [SDS], 2% β-mercaptoethanol, 192 mmol/L glycine, 25 mmol/L Tris, and 5% sucrose), heated for 5 min at 100°C, then centrifuged at 12,000× *g* for 15 min. The pellets were discarded, and the supernatants were run on a 15% SDS–polyacrylamide gel (SDS-PAGE) before transfer to nitrocellulose membrane and immunoblotting with anti-PrP antibodies. Western blot methods used to identify and characterize PrP^res^ in TgOvPrP4 mice have been previously described (*19*,*20*). Briefly, PrP^res^ was obtained after mouse brain homogenates were treated with proteinase K (Roche, Meylan, France) (10 µg/100 mg brain tissue for 1 h at 37°C) and concentration by ultra-centrifugation (100,000 *g* for 2 h on a 10% sucrose cushion). After denaturation in TD4215 buffer, PrP^res^ was separated in 15% SDS-PAGE, electroblotted to nitrocellulose membranes, then detected on the membrane by using anti-PrP monoclonal antibodies.

PrP^res^ was detected by using anti-PrP Bar233 monoclonal antibody (ascitic fluid 1/10,000, provided by J. Grassi) or Sha31 (1/10 from kit TeSeE sheep/goat Bio-Rad) against the ovine 152-FGSDYEDRYYRE-163 and 156-YEDRYYRE-163 PrP sequences, respectively. Quantitative studies of PrP^res^ polypeptide molecular mass and glycoforms proportions were performed by using Quantity One (Bio-Rad) software analysis of chemiluminescent signals following >3 independent runs of the samples from >3 different mice per experimental group. Glycoforms ratios were expressed as mean percentages (± standard deviations) of the total signal for the 3 PrP^res^ glycoforms and the apparent molecular masses were evaluated by comparison of the positions of the PrP^res^ bands with a biotinylated marker (B2787; Sigma, St. Quentin Fallavier, France).

### Histopathologic Analyses

Mouse brain fixed in buffered 4% paraformaldehyde was treated for 1 h at room temperature with formic acid (98%–100%) before being embedded in paraffin blocks (Thermo Electron, Cergy-Pontoise, France). Tissue sections, 5 μm thick, were cut from paraffin blocks, placed on treated glass slides (Starfrost, Medite Histotechnic, Burgdorf, Germany), and dried overnight at 55°C. Once dewaxed, slides were stained for either histopathologic or immunohistochemical examination. Amyloid deposits were identified with a Congo red stain, and vacuolar lesions were observed on slides stained with hematoxylin-eosin (HE), according to Fraser’s lesion profile analyses (*26*). Lesion profiles were measured by using a computer-assisted method (*27*). Brain slices were immunostained for the presence of disease-associated prion protein (PrP^d^) by using 2 μg/mL of anti-PrP SAF84 monoclonal antibody (SPI Bio, Massy, France). Recently described pre-treatments designed to enhance PrP^d^ detection were also applied (*28*). These consisted of a 10-min formic acid (98%) bath at room temperature, 20 min of hydrated autoclaving at 121°C (Prestige Medical, AES Laboratories, Blackburn Lane, UK), and digestion at 37°C with proteinase K (Roche Diagnostics, Meylan, France) at a concentration of 20 μg/mL for 15 min, with an additional incubation with streptomycin sulfate at 8.75 mmol/L for 1 h. Endogenous peroxidase activity was also blocked. A peroxidase-labeled avidin-biotin complex (Vectastain Elite ABC, Vector Laboratories, Burlingame, CA, USA) and a solution of diaminobenzidine intensified with nickel chloride (DAB-Ni, Zymed, Montrouge, France) to give black deposits were used to amplify and visualize binding of the disease-associated form of PrP (PrP^d^). Final detection was achieved with a solution of diaminobenzidine intensified with nickel chloride (DAB-Ni, Zymed) to give black deposits. The specificity of PrP^d^ immunolabeling was also assessed by using uninfected brain samples. Finally, the slides were counterstained with aqueous hematoxylin, dehydrated, mounted by using Eukitt mounting medium (VWR International, Limonest, France) and observed under a light microscope BX51 (Olympus, Rungis, France) coupled to an image analysis workstation (MorphoExpert Software, Explora Nova, La Rochelle, France).

## Results

### Transmission of TSE Isolates in TgOvPrP4 Mice

To determine the ability of the 4 bovine TSE isolates (experimental bovine TME, L-type BSE, H-type BSE, and a typical BSE natural isolate) to cause a TSE in a common host species, they were inoculated into TgOvPrP4 mice (Table). The shortest survival period was observed in mice infected with the typical BSE isolate (421 ± 48 days), compared with 436 ± 77 days or 627 ± 74 days in TgOvPrP4 mice inoculated with bovine TME or L-type BSE, respectively. At second serial passage of bovine TME, L-type BSE, and typical BSE into TgOvPrP4 mice, the survival periods were shortened for all 3 sources, but especially for L-type BSE (202 ± 26 days) and bovine TME (234 ± 27 days), although survival periods were statistically different for these 2 groups of mice (p = 0.0095). The survival period for typical BSE (354 ± 48 days) was significantly longer than that found for L-type BSE and bovine TME (p<0.0001). For the ovine BSE isolate, the incubation period was 296 ± 46 days at first passage, but the period increased to 365 ± 36 days at second passage. The survival period at second passage of ovine BSE was not significantly different from that of typical BSE (p = 0.814). Most of the mice inoculated with the 3 bovine TSE isolates were positive (28/31 at first passage) for PrP^res^ by Western blot or PrP^d^ by immunohistochemical testing. In contrast, all (8/8) the transgenic mice inoculated with the H-type BSE isolate, which had a survival period of 692 ± 129 days, were negative for PrP^res^ by either Western blot or PrP^d^ by immunohistochemical analyses.

**Table:** TSE sources transmitted to TgOvPrP4 mice*

TSE sources	Nature	First passage			Second passage	
		Survival periods (mean ± SD dpi)	No. PrP^d^ positive mice/total†		Survival periods (mean ± SD dpi)	No. PrP^d^ positive mice/total†
Experimental isolates						
TME	TME	436 ± 77	9/11		234 ± 27	9/9
SB1	Ovine BSE	296 ± 46	19/19		365 ± 36	11/12
Natural isolates						
02-2528	L-type BSE	627 ± 74	9/10		202 ± 26	9/9
03-2095	H-type BSE	692 ± 129	0/8		ND	ND
01-2281	Typical BSE	421 ± 48	10/10		354 ± 48	10/10

*TSE, transmissible spongiform encephalopathy; dpi, days postinoculation; PrP^d^, disease-associated prion protein; TME, transmissible mink encephalopathy; BSE, bovine spongiform encephalopathy; ND, not done**.** †Results obtained by Western blot or by immunohistochemical test.

### PrP**^res^** Molecular Features in Cattle and in TgOvPrP4 Mice

**Figure 1:**
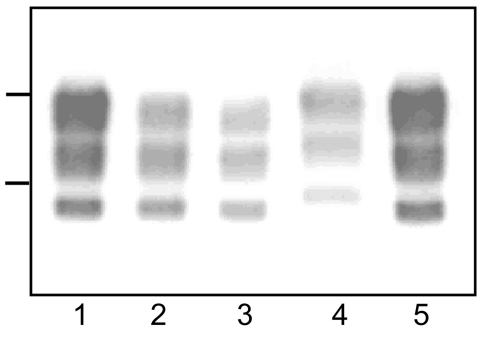
Western blot analyses of protease-resistant prion protein from proteinase K–treated brain homogenates from cattle transmissible spongiform encephalopathies (TSEs). Typical bovine spongiform encephalopathy (BSE) (lanes 1, 5), L-type BSE (lane 2), transmissible mink encephalopathy (TME) in cattle (lane 3), H-type BSE (lane 4). Bars to the left of the panel indicate the 29.0- and 20.1-kDa marker positions.

**Figure 2:**
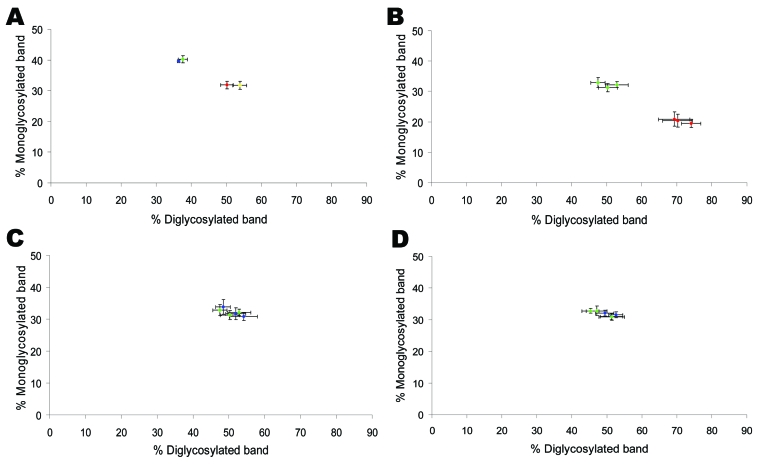
Glycoforms proportions (means ± standard deviations) of protease-resistant prion protein detected by using Sha31 antibody. A) Cattle transmissible spongiform encephalopathies (TSEs). B) First passage of L-type and typical bovine spongiform encephalopathy (BSE) into TgOvPrP4 mice. C) First passage of transmissible mink encephalopathy (TME) in cattle and L-type BSE into TgOvPrP4 mice. D) Second passage of TME in cattle and L-type BSE into TgOvPrP4 mice. Results from 3 individual mice from each experimental group are shown. Typical BSE in red, H-type BSE in yellow, L-type BSE in green, and TME-in-cattle in blue.

Western blot analysis of PrP^res^ from bovine TME, L-type BSE, H-type BSE, and typical BSE used for inoculation into TgOvPrP4 mice was performed to compare the molecular features of PrP^res^. The bovine TME and L-type BSE isolates had similar molecular masses for the 3 PrP^res^ polypeptides (Figure 1). The unglycosylated PrP^res^ polypeptide has a similar molecular mass (≈18.5 kDa) in L-type BSE, bovine TME, and in typical BSE, whereas a difference in molecular mass (≈0.5–0.8 kDa lower) was found for the diglycosylated band in both L-type BSE and bovine TME compared to typical BSE. A comparison of PrP^res^ glycoform ratios also showed similar proportions of the diglycosylated and monoglycosylated isoforms of PrP^res^ in bovine TME and L-type BSE, both of which had lower levels of diglycosylated PrP^res^ than in typical BSE (Figure 2, panel A).

**Figure 3:**
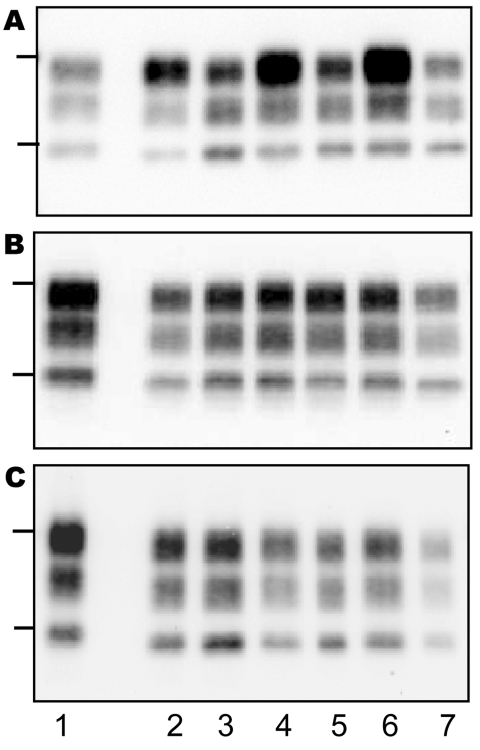
Western blot of protease-resistant prion protein from TgOvPrP4 mice after proteinase K digestion and immunodetection with anti-PrP Sha31 antibody. A) First passage of typical bovine spongiform encephalopathy (BSE) (lanes 2, 4, and 6) and L-type BSE (lanes 3, 5, and 7). B) First passage of TME in cattle (lanes 2, 4, and 6) and L-type (lanes 3, 5, and 7). C) Second passage of TME in cattle (lanes 2, 4, and 6) and L-type BSE (lanes 3, 5, and 7). Each lane shows PrP^res^ from a distinct individual mouse from each experimental group. Bars to the left of the panel indicate the 29.0- and 20.1-kDa marker positions. Lane 1, PrP^res^ control from a scrapie-infected TgOvPrP4 mouse (C506M3 strain).

On transmission of the bovine TSE isolates to TgOvPrP4 mice, the L-type BSE differed from typical BSE in its lower proportion of diglycosylated PrP^res^, but the molecular masses of the unglycosylated PrP^res^ polypeptides were similar between these isolates (Figure 2, panel B, and Figure 3, panel A). In contrast, Western blot analysis of PrP^res^ showed indistinguishable patterns in transgenic mice infected with the L-type BSE or bovine TME, with respect to the molecular mass of the 3 PrP^res^ polypeptides (Figure 3, panel B) and the ratio of these PrP^res^ glycoforms (Figure 2, panel C). These comparable features were maintained in both L-type BSE and bovine TME at second passage in TgOvPrP4 mice (Figure 2, panel D, and Figure 3, panel C), and these were distinct from typical BSE, on the basis of the ratio of the 3 PrP^res^ glycoforms.

### Histopathologic Features of TME in Cattle and L-type BSE in TgOvPrP4 Mice

**Figure 4:**
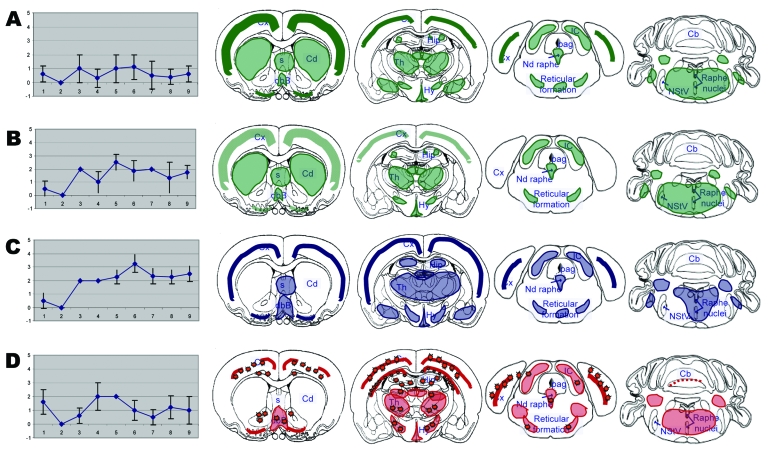
Brain lesion profiles (left panels) and disease-associated prion protein brain distribution (right panels) observed in the brain of TgOvPrP4 mice infected with L-type bovine spongiform encephalopathy (BSE), at first (A, n = 5) and second (B, n = 4) passages; TME in cattle (C, n = 5); or typical BSE (D, n = 4) at second passage. Brain vacuolation was scored (means ± standard deviations) on a scale of 0–5 in the following brain areas: 1) dorsal medulla nuclei, 2) cerebellar cortex, 3) superior colliculus, 4) hypothalamus, 5) central thalamus, 6) hippocampus, 7) lateral septal nuclei, 8) cerebral cortex at the level of thalamus, and 9) cerebral cortex at the level of septal nuclei. In right panels, showing the PrP^d^ distribution, stars indicate the presence of florid plaques.

To further examine the phenotypes of the bovine TSE agents, the distribution of vacuolar lesions and the distribution and features of PrP^d^ were investigated at standardized brain levels of TgOvPrP4 mice (Figure 4). TgOvPrP4 mice infected with L-type BSE at first passage showed low vacuolar lesion intensity but PrP^d^ accumulation was strongly detected, which was characterized by PrP^d^ aggregation into plaques. These plaques were not amyloid based on an absence of Congo red birefringence (data not shown). At the second passage, L-type BSE agent induced a lower degree of PrP^d^ accumulation than in the first passage; the type of deposition was fine powdery to granular, and no plaques were observed. Although the brain lesion profile showed a higher degree of vacuolation than in the first passage, the PrP^d^ mapping was similar at first and second passage in transgenic mice. At second passage, some additional sites had PrP^d^ accumulation, including the septal areas and the midbrain. The L-type BSE remained distinct from typical BSE, in terms of lesion profiles and types of PrP^d^ deposition. In the brain of TgOvPrP4 mice infected with typical BSE and ovine BSE, numerous florid plaques containing PrP^d^ were seen, and these were amyloid, based on Congo red staining. The florid plaques were prominent in the cortical regions of the brain in transgenic mice infected with typical BSE but were not found in any of the mice infected with L-type BSE at either first or second passage. At second passage, vacuolar changes were more intense in the dorsal medulla nuclei, hypothalamus, and hippocampus in mice infected with typical BSE; in L-type BSE-infected mice, lesions were more pronounced in the colliculus, thalamus, and cerebral cortex.

The distribution of vacuolar changes and PrP^d^ in the brain of TME-infected mice was similar to that observed in mice infected with L-type BSE, although minor differences in the PrP^d^ immunolabeling intensities were found in some brain regions, including the absence of the fine, powdery PrP^d^ in the caudate putamen of bovine TME-infected mice. It is noteworthy that the PrP^d^ distribution was also comparable in transgenic mice infected with bovine TME and typical BSE; however, no florid plaques were detected in any of the mice infected with bovine TME.

## Discussion

Comparison of TME and 3 distinct types of BSE in a common host species was prompted by previous observations that TME, whose origin is unknown, did not reduce its pathogenicity for mink after passage into cattle (*8*), and transmission of TME into cattle resulted in a low-molecular-mass PrP^res^ profile (*29*). A similar low-molecular-mass PrP^res^ profile has been observed in L-type BSE or BASE (*11*,*13*,*16*), and in the current study, these similar PrP^res^ molecular properties between bovine TME and L-type BSE were demonstrated in TgOvPrP4 mice. These 2 distinct bovine TSE sources were both readily transmitted into TgOvPrP4 mice (illustrating the usefulness of transgenic mouse models for prion agent strain typing when transmission to a common wild-type rodent is not possible) and had several common features including survival periods, PrP^res^ molecular features, and the distribution of vacuolar pathologic changes. This combination of biochemical and phenotypic properties indicates that they have similar biologic properties in TgOvPrP4 mice and could represent independent isolation of the same TSE strain, although some subtle histologic differences between L-type BSE and bovine TME were observed on second passage in TgOvPrP4 mice. Additional serial passages from these cattle TSE sources are required for further characterization, especially since the possibility cannot be excluded that transmission of a cattle TSE into mink can modify its biologic properties.

In contrast, transmission of L-type BSE and typical BSE into TgOvPrP4 mice resulted in different incubation periods, PrP^res^ molecular properties, and histopathologic features during 2 serial passages. The survival period at second passage for L-type BSE was considerably reduced and significantly shorter (>120 days) than typical BSE after 2 passages into TgOvPrP4 mice. Although florid plaques were found in transgenic mice infected with typical BSE, they were not observed in mice following first or second passage of L-type BSE in TgOvPrP4 mice. Analysis of PrP^res^ properties also found differences between L-type and typical BSE with respect to the relative proportion of diglycosylated PrP^res^ polypeptides. The lower molecular mass of the unglycosylated PrP^res^ polypeptide in L-type BSE was maintained in TgOvPrP4 mice as well as in typical BSE. In cattle, the size of the unglycosylated PrP^res^ polypeptide in L-type BSE is within the range of that found in typical BSE (*16*). Notably, our findings did not produce evidence for modification of the phenotypic features of these cattle TSEs on passage into TgOvPrP4 mice, which was recently described after transmission of L-type BSE into wild-type (*18*) or tg338 ovine transgenic (*24*) mice.

The current and previous studies demonstrate that when typical BSE and L-type BSE are transmitted into TgOvPrP4 mice, key features of these cattle TSEs were maintained (*19*–*21*). However, these transgenic mice were not susceptible to the H-type BSE, as confirmed with 4 other isolates (data not shown). This finding is in contrast to a previous study that demonstrated transmission of H-type BSE into a different transgenic mouse line expressing the ovine prion protein gene (*15*). Possible explanations for this discrepancy are the lower level of the cellular prion protein in TgOvPrP4 mice compared with tg338 mice (2- to 4-fold vs. 8- to 10-fold greater than ovine brain), different cellular patterns of expression due to the use of different promoters, or the different sequence of the prion gene (V_136_R_154_Q_171_ in tg338 mice) (*20*,*30*). These findings on the transmission of H-type into transgenic mice provide further evidence for the distinct biologic properties of this cattle TSE compared to L-type BSE and typical BSE.

The Stetsonville isolate of TME that was experimentally passaged into cattle was also readily transmitted into TgOvPrP4 mice and resulted in a TSE phenotype that shared common biologic features with L-type BSE, but not typical BSE, in these transgenic mice. Transmission of TME from temporally and geographically different outbreaks into cattle showed that bovine TME is similar among the different isolates but all of them are phenotypically distinct from typical BSE (*7*). Histopathologic studies showed more severe spongiform changes, especially at rostral levels, in cattle infected with TME (*7*) than infected with typical BSE, and these changes were also observed in TgOvPrP4 mice infected with L-type BSE at second passage. Conversely, mink infected with typical BSE had a greater degree of spongiform change in the brainstem than mink infected with TME (*10*). Similar changes were also observed in TgOvPrP4 mice infected with typical BSE. This preferential involvement of rostral brain regions in L-type BSE has also been described in cattle (*13*) and on transmission into bovine transgenic mice (*18*,*24*). Transmission of TME or typical BSE into wild-type mice (*31*,*32*) or hamsters (*33*,*34*) also resulted in distinct transmissibility between these TSEs in these host species. Based on these findings, we conclude that typical BSE is not a likely source for TME in mink; however, if TME were to be due to infection with a cattle TSE, the most likely candidate is L-type BSE.

L-type BSE has not been reported in the United States, although importation of cattle or cattle products with a TSE infection cannot be excluded as a potential source. Recently, 2 BSE cases identified in cattle born in the United States had unusual PrP^res^ properties similar to those described for H-type BSE (*11*,*12*,*35*,*36*), which raised the possibility that atypical TSEs in cattle may be a source for TME infection. In Europe, the prevalence of the H-type or L-type BSE is estimated to be very low (in France the L-type BSE occurs at a prevalence of 1 case found PrP^res^ positive in the brain stem per 3 million cattle tested per year) (*23*). The very rare prevalence of TME may be partially due to the rare occurrence of cattle TSEs that enter the mink diet (*8*,*29*).

## Conclusion

These studies provide experimental evidence that the Stetsonville TME agent is distinct from typical BSE but has phenotypic similarities to L-type BSE in TgOvPrP4 mice. Our conclusion is that L-type BSE is a more likely candidate for a bovine source of TME infection than typical BSE. In the scenario that a ruminant TSE is the source for TME infection in mink, this would be a second example of transmission of a TSE from ruminants to non-ruminants under natural conditions or farming practices in addition to transmission of typical BSE to humans, domestic cats, and exotic zoo animals (*37*). The potential importance of this finding is relevant to L-type BSE, which based on experimental transmission into humanized PrP transgenic mice and macaques, suggests that L-type BSE is more pathogenic for humans than typical BSE (*24*,*38*).
